# p53 as a unique target of action of cisplatin in acute leukaemia cells

**DOI:** 10.1111/jcmm.17502

**Published:** 2022-08-09

**Authors:** Sanjay Kumar, Paul B. Tchounwou

**Affiliations:** ^1^ Cellomics and Toxicogenomics Research Laboratory, NIH/NIMHD‐RCMI Center for Health Disparities Research Jackson State University Jackson Mississippi USA; ^2^ Department of life Sciences, School of Earth, Biological, and Environmental Sciences Central University South Bihar Gaya India

**Keywords:** acute promyelocytic leukaemia, cisplatin, CRISPR‐Cas9, MDM2‐DAXX‐HAUSP, p53

## Abstract

Acute promyelocytic leukaemia (APL) occurs in approximately 10% of acute myeloid leukaemia patients. Arsenic trioxide (ATO) has been for APL chemotherapy, but recently several ATO‐resistant cases have been reported worldwide. Cisplatin (CDDP) enhances the toxicity of ATO in ovarian, lung cancer, chronic myelogenous leukaemia, and HL‐60 cells. Hence, the goal of this study was to investigate a novel target of CDDP action in APL cells, as an alternate option for the treatment of ATO‐resistant APL patients. We applied biochemical, molecular, confocal microscopy and advanced gene editing (CRISPR‐Cas9) techniques to elucidate the novel target of CDDP action and its functional mechanism in APL cells. Our main findings revealed that CDDP activated p53 in APL cells through stress signals catalysed by ATM and ATR protein kinases, CHK1 and CHK2 phosphorylation at Ser 345 and Thr68 residues, and downregulation and dissociation of MDM2‐DAXX‐HAUSP complex. Our functional studies confirmed that CDDP‐induced repression of MDM2‐DAXX‐HAUSP complex was significantly reversed in both nutilin‐3‐treated KG1a and p53‐knockdown NB4 cells. Our findings also showed that CDDP stimulated an increased number of promyelocytes with dense granules, activated p53 expression, and downregulated MDM2 in liver and bone marrow of APL mice. Principal conclusion of our study highlights a novel mode of action of CDDP targeting p53 expression which may provide a basis for designing new anti‐leukaemic compounds for treatment of APL patients.

## 1 INTRODUCTION

Acute promyelocytic leukaemia (APL) accounts for approximately 10% of acute myeloid leukaemia (AML) cases (~600–800 cases per year) in the United States. APL has well‐defined clinical and pathologic features. APL is classified as the M3 subtype of AML in the French American British system and classified as APL with t(15;17) (q22;q12), promyelocytic leukaemia–retinoic acid receptor‐alpha (PML–RARα) by the WHO.^1^ APL represents about 10% of AML in the United States, accounting for about 600–800 cases per year.^1^, ^2^ It is known as the M3 type of AML that is formed as a result of translocation mutation involving chromosomes 15 and 17 inside the bone marrow. As a consequence, two fusion oncogenes (PML–RARα and RARα–PML) are formed.^1^, ^2^ These oncogenes are responsible for APL pathogenesis and are also used in diagnosis and monitoring of APL patients.^3^, ^4^ Arsenic trioxide (ATO) alone and/or combination with all trans retinoic acid (ATRA) have been used successfully for the cure of APL patients who have PML–RARα oncogene, providing a complete treatment and maximizing the survival rate of patients.^2^, ^4^, ^5^ However, chemoresistance of both ATO and ATRA combination has been reported in APL patients who do not have the PML–RARα oncogene but possess the X–RARα oncogenes.^6^

Cisplatin is an anti‐tumour drug^7^ that has been successful utilized in the cure of various types of human neoplasms.^2^, ^8^ Scientific evidence suggests that CDDP enhances the toxicity of ATO in the cancer cells of head and neck^9^ and lung^10^ and may be a better option to help X‐RARα APL patients who show resistance to ATO resistance treatment. The tumour suppressor protein, p53, has been shown to play a key role in the regulation of cell cycle and the induction of apoptosis in cells undergoing various typed of stress conditions.^11^, ^12^ However, it has also been demonstrated that the mouse double minute 2 (MDM2) and MDM4/MDMX predominantly controlled p53 expression by inducing proteasomal degradation and ubiquitylation in normal living cells.^13^

Scientific evidence has also shown that MDM2 negatively regulates p53 activity and is a very unstable protein that usually remains associated with adapter protein, death‐associated protein 6 (DAXX) through deubiquitinating enzyme, and herpesvirus‐associated ubiquitin‐specific protease (HAUSP), resulting in the formation of MDM2‐DAXX‐HAUSP complex that acts together to prevent self‐ubiquitination and degradation.^13^, ^14^ MDM2‐DAXX‐HAUSP complex is a prominent target of several anticancer drugs, and its degradation in cancer cells leads to p53 activation, cell cycle arrest, and programmed cell death.^4^, ^13^, ^14^, ^15^, ^16^ P53 is novel target of APL patients treatment strategy, and accumulating evidence suggests that the PML gene interacts with p53 and causes apoptosis in APL cells.^17^

It has also been reported that the expression of both p53 and p21 is downregulated in cancer cells where promyelocytic leukaemia zinc finger‐retinoic acid receptor α (PLZF‐RARα) stimulates cancer cell growth in APL patients.^18^ From an experimental study using a mouse model of APL, scientific evidence has shown that the PML‐transformation–related protein (Trp53) is involved in eradication of leukaemia–initiating cells.^19^ Pseudokinase Tribble 3 (TRIB3) suppresses p53‐mediated senescence and stimulates APL cell proliferation.^20^ Using acute lymphoblastic leukaemia cells as a test model, it has also been demonstrated that Nutilin‐3 reduces the expression of MDM2 and activates p53, leading to apoptosis.^21^, ^22^

Cisplatin acts in several ways inside APL patients to inhibit cell growth and trigger cell mortality. Few studies have revealed that CDDP inhibits cell cycle progression,^23^ induces oxidative damage, and causes programmed death of HL‐60 cells by reducing Bcl2 expression and stimulating BCL_2_L12 protein.^24^ CDDP also regulates the activity of c‐jun and PKC signalling cascade to DNA damage and apoptosis in leukaemia cells.^25^ Accumulating evidence suggests that it produces cytotoxicity through interaction with DNA, RNA, and proteins of cells. CDDP works inside the cell through several molecular mechanisms including DNA‐adduct interaction, induction of oxidative damage, alteration of DNA synthesis and cell cycle regulation, and stimulation of programmed cell death.^2^, ^23^, ^24^, ^25^, ^26^ It has also been reported that CDDP toxicity is associated with upregulation of caspase 3 activity in CEM leukaemia cells,^27^ and DNA‐adduct formation in both mouse leukaemia L1210 cells^28^ and APL cells.^2^ P53 also modulates cell cycle progression, genomic stability, and programmed death of a variety of cancer cells. It may be mutated, deleted, or suppressed by several ubiquitin ligases (E3) in most of human cancer cells.^13^, ^29^

Reactivation of p53 by therapeutic agents in cancer cells could be an exciting new strategy to treat cancer.^30^ Since CDDP activates p53 in APL cells,^2^ an investigation of its molecular mechanism targeting the p53 pathway would lead to the identification of a new target of therapeutic action. We have discovered from this research that CDDP‐induced p53 activation is mediated through stress signal transmission, interaction with DAXX, dissociation of MDM2‐DAXX‐HAUSP, and increased number of promyelocytes with dense granules in bone marrow cells. This signalling mechanism leads to an arrest in cell cycle arrest and subsequently to a programmed death of APL cells. CDDP’s novel mode of action targeting p53 activation may provide a basis for designing new anti‐leukaemic compounds for treatment of APL patients.

## 2 MATERIALS AND METHODS

### 2.1 Cell line and culture

HL‐60, NB4, and KG‐1a cells were used in this study. The cells were purchased from the American Type Culture Collection (ATCC) and National Cell Culture Center (NCCC), and Biovest International, Inc. and maintained according to standard procedures (5% CO_2_ at 37°C). These three cell lines were chosen based on ATCC and NCCC fact sheets and on the fact that many previously published articles have used them as test models for APL research.

### 2.2 Reagents

Cisplatin (cat # 141398) was purchased from Abcam company. Nutlin‐3 and protease inhibitor was obtained from Sigma‐Aldrich. Anti‐DAXX, anti‐HAUSP was purchased by Cell Signaling Technology. Anti‐MDM2 is purchased from Santa Cruz Biotechnology Inc. Hoechst 33342, Alexa fluor 568, and Alexa fluor 568 were purchased from Life Technologies.

### 2.3 WST cell proliferation assay

Acute promyelocytic leukaemia cell lines (5 × 10^4^ HL‐60/KG1a/NB4 cells/well) were grown in the absence or presence of different concentrations (5, 10, 20, 40, and 80 μM) of CDDP in 100 μl culture medium for 48 h. After incubation, WST cell proliferation was performed by Abcam Company (Cat # ab65473) instruction manual. In brief, after cisplatin treatment, 10‐μl WST/ECS solution was added to each well and further incubated for 4 h at 37°C. After incubation, absorbance in each well was measured absorbance at 440 nm using a microtiter plate reader and the percentages of cell proliferation of all samples were calculated.

### 2.4 Immunoprecipitation and Western blotting

After treatment with different concentrations of CDDP (5, 10, 20, 40, and 80 μM) to APL cells, protein lysates were prepared in RIPA buffer by centrifugation of cells, washed with phosphate buffer saline (PBS), and sonication. We also treated APL mice with different doses (1, 2, 4, 6, and 8 mg/kg body wt.) of cisplatin and prepared liver protein lysates in RIPA buffer. We used 500ug protein lysate of APL cells/liver tissue lysate of each sample. Western blotting and immunoprecipitation (IP) were performed, as described earlier.^2^, ^4^

### 2.5 Immunocytochemistry and confocal microscopy imaging

NB4 cells (1 × 10^5^) were cultured in the presence or absence of CDDP and attached on poly‐l‐lysine coated slides. Immunocytochemistry of attached cells was performed using Ki67 antibody (dilution, 1:100) (cat# 33–4711) or p53 antibody (cat # 9282) and PML‐RARα (cat# ab43152) from Life Technology, Cell Signaling or Abcam company and imaged by confocal microscopy (Olympus Company), as described earlier.^2^

### 2.6 Cell cycle analysis

NB4 cells were treated with different concentrations (5, 10, 20, 40, and 80 μM) of CDDP for 48 h at 37°C. After incubation, cells were washed with PBS and fixed in 100% cold ethanol on ice for 15 min. Fixed cells were centrifuged at 400 *g* and removed ethanol completely. Cells were stained with propidium iodide (PI) (50 μg/ml) at 37°C for 40 min and analysed by flow cytometry (BD FACS Calibur), as described earlier.^31^, ^32^

### 2.7 Apoptosis assay

NB4 cells (1 × 10^6^) were treated with different concentrations of CDDP for 48 h. After treatment, both early and late apoptosis were assayed using the Alexa Fluor 488 Annexin V/Dead cell Apoptosis kit (Invitrogen; Cat. No. V13241). In brief, both control and CDDP‐treated cells were washed with ice cold PBS and incubated 15 min in mixture of annexin V and PI at room temperature. After incubation, cells were washed with 1X binding buffer and apoptosis analysed using the Cellometer Vision CBA/confocal microscopy (Fluoview 10i, Olympus), as previously described.^33^

### 2.8 Pulse‐chase assay

The turnovers of protein of complex molecules were investigated in NB4 cells by using a protein synthesis inhibitor cycloheximide (CHX) standard assay. In brief, cells were treated with 50 μg/ml CHX along with different concentration (5, 10, 20, 40, and 80 μM) of CDDP for 48 h and the expression level of p53, and MDM2 was analysed by Western blotting as previously described.^14^

### 2.9 Immunohistochemistry (IHC)

We collected livers in RIPA buffer of both untreated/controls and APL mice treated with different doses (1, 2, 4, 6 and 8 mg/kg body wt). Liver tissues were frozen in embedding medium (Polarstat Plus), and 5 μM sections were made using Cryostar NX50 (Thermo Scientific). Liver sections were fixed in acetone and methanol mixture at −20°C for 5 min and permeabilized with 0.2% triton X at 4°C for 10 min. They were washed three times with PBS and blocked in 5% normal goat serum containing 4% BSA for 30 min at room temperature. Blocked sections were incubated in anti‐p53 (1:100) and anti‐MDM2 (1:100) antibodies inside humidified chamber for 4 h at room temperature. Again, the sections were washed three times with PBS and further incubated secondary antibody [Alexa fluor 488 & 594 (11000)] for 1 h at room temperature. After incubation, sections were washed with PBS and the images were captured under fluorescence microscope, IX73 (Olympus), and presented as shown previously.^34^

### 2.10 Knockdown of p53 in NB4 cells

We made p53 knockdown NB4 cells using a lentivirus shRNA (Dharmacon Inc) method, as previously described.^35^ In brief, we seeded 10,000 NB4 cells in 25 μl of transduction medium (RPMI 1640) without serum in each well with polybrene (8 μg/ml). Then, we added 40 MOI SMART choice lentiviral p53 shRNA particles (10^5^ TU/μl) to each well and incubated for 20 h at 37°C. After incubation, we added 75 μl of 20% serum containing culture medium in each well and further cultured for 2 days at 37°C. We performed microscopic examination and cell viability test and further incubated with puromycin (8 μg/ml) for a week. Puromycin‐selected NB4 cells were further checked through western blotting and fluorescence imagining. We used for our experiment more than 90% p53 knock‐down NB4 cells.

### 2.11 Transgenic APL mice

Acute promyelocytic leukaemia transgenic mice were purchased from The Jackson Laboratory in Bar Harbor, Maine, USA. Our APL transgenic mice strain is C57BL/6‐Pml^tm1(PML/RARA)Ley^/J and Stock No: 017959 having gene construct, promyelocytic leukaemia‐retinoic acid receptor alpha (PML‐RARα) widely expressed fusion proteins and tag with Cre recombinase. APL transgenic mice were kept in the Animal Core Facility following the guidelines and recommendations of Jackson State University IACUC committee. After 1 month of acclimatization, they were bred to produce enough mice for experimentation. Regular genotyping was done from young pups mice tail blood and proper homozygous mice containing our desired oncogene (PML‐RARα) responsible for pathogenesis of APL was performed.^36^ Young transgenic mice (8–12 weeks old with average weight of 20–30 g) were used for this experiment. Each treatment group was made of five young mice of similar weight and age. They were treated with five doses of cisplatin (1, 2, 4, 6, and 8 mg/kg body wt) for 3 days by a continuous intraperitoneal treatment based on previous publication^37^ and our standardization of procedure. After 3 days of CDDP treatment of transgenic mice, the liver tissue was dissected and the bone marrow cells were isolated for further experimentation.

### 2.12 Bone marrow isolation from transgenic mice

Young transgenic mice (8–12 weeks old with average weight of 20–30 g) were treated with different doses of cisplatin (1, 2, 4, 6, and 8 mg/kg body wt) for 3 days continuously. After treatment, the transgenic mice were euthanized through CO_2_ asphyxiation and bone marrow cells were isolated from the femur and tibia, as described previously.^38^

### 2.13 Wright staining of promyelocytes inside the bone marrow cells

We made thin smear of sterile bone marrow cells of all dose cisplatin treated or untreated mice samples on glass slides and promyelocytes stained with Wright‐Giemsa solution, as described previously.^39^ In brief, air dry bone marrow cells smear was placed in Wright‐Giemsa solution for about 3 min. After incubation, slides were dipped in phosphate buffer (pH = 7.2) for 10 min. Then, slides were rinsed with distilled water and air dry, and the stained promyelocytes images were taken using the Arcturus Laser Capture Micro (LCM) dissection system.

### 2.14 Statistical analysis

Experiments were performed in triplicates. Data were presented as means +/− SDs. When appropriate, one‐way ANOVA or Student paired t‐test was performed using SAS software available in the Biostatistics Core Laboratory at Jackson State University. *p*‐values less than 0.05 were considere statistically significant.

## 3 RESULTS

### 3.1 CDDP inhibits APL cell proliferation

**FIGURE 1:**
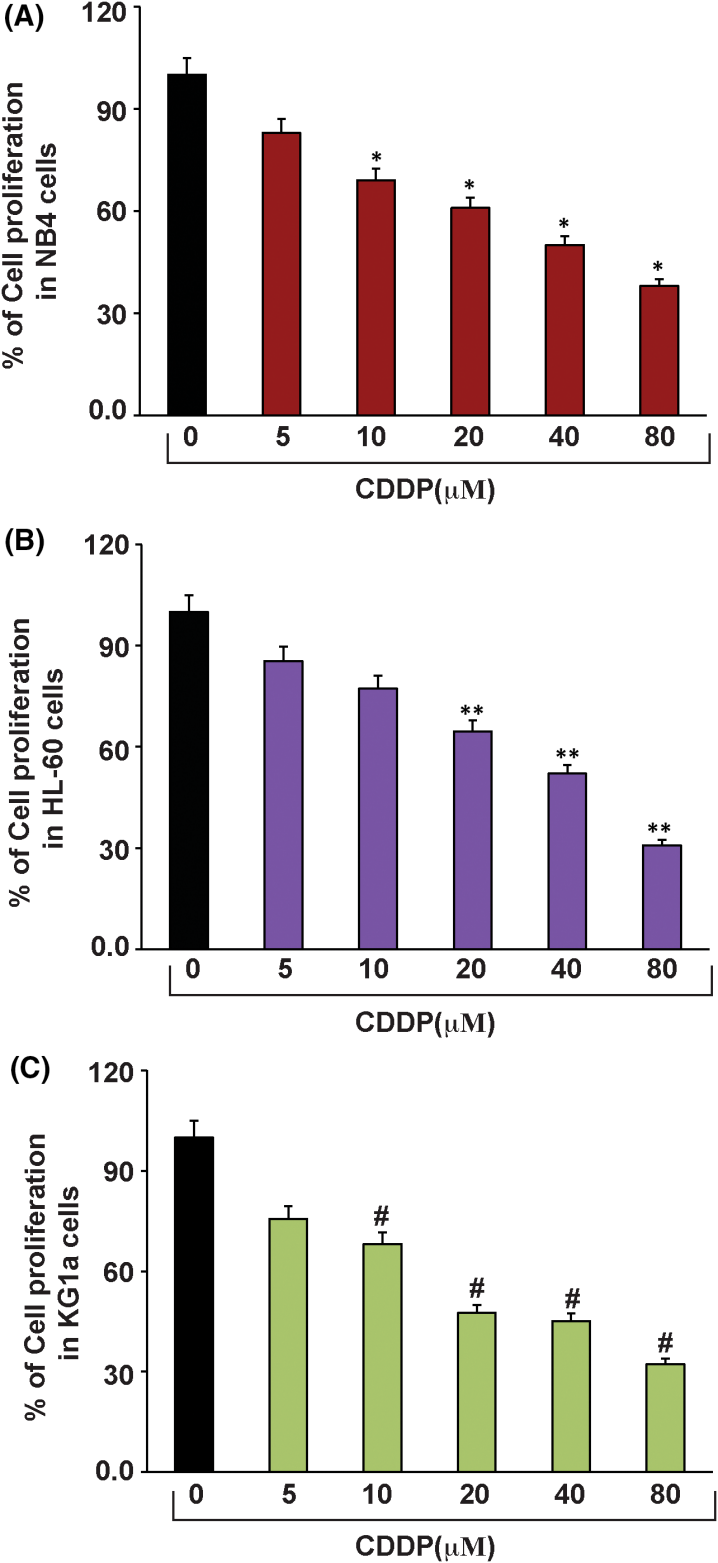
CDDP inhibits APL cell proliferation. APL (HL‐60, KG‐1a and NB4) cells were treated with different concentrations (0, 5, 10, 20, 40, and 80 μM) of CDDP for 48 h and further incubated for 4 h in WST/ECS solution. After incubation, absorbance in each well was measured using a microplate reader and the percentages of cell viability were presented with respect to CDDP concentrations. Data represent the means of three independent experiments **±** SDs (*p < 0.01) (A), (**p < 0.01) (B) and (#*p* < 0.01) (C). Highly statistically significant inhibition (*p* < 0.01) in cell proliferation was observed in NB4 (A), HL‐60 (B), and KG‐1a (C).

To assess the inhibitory action of CDDP on APL cells, we used the Abcam Company WST‐1 cell proliferation protocol. We treated the cells with CDDP concentrations of 0, 5, 10, 20, 40, and 80 μM for a period of 48 h. Cells were then treated them with tetrazolium salt WST‐ 1 for 4 h, and the amount of formazan dye produced was quantified using a microtiter plate reader. The study results showed that CDDP treatment produced a statistically significant reduction (*p* < 0.05) in the viability of APL cells, especially at the 20, 40, and 80 μM concentrations (Figure 1A–C).

### 3.2 CDDP activates p53 expression and cell cycle arrest

**FIGURE 2:**
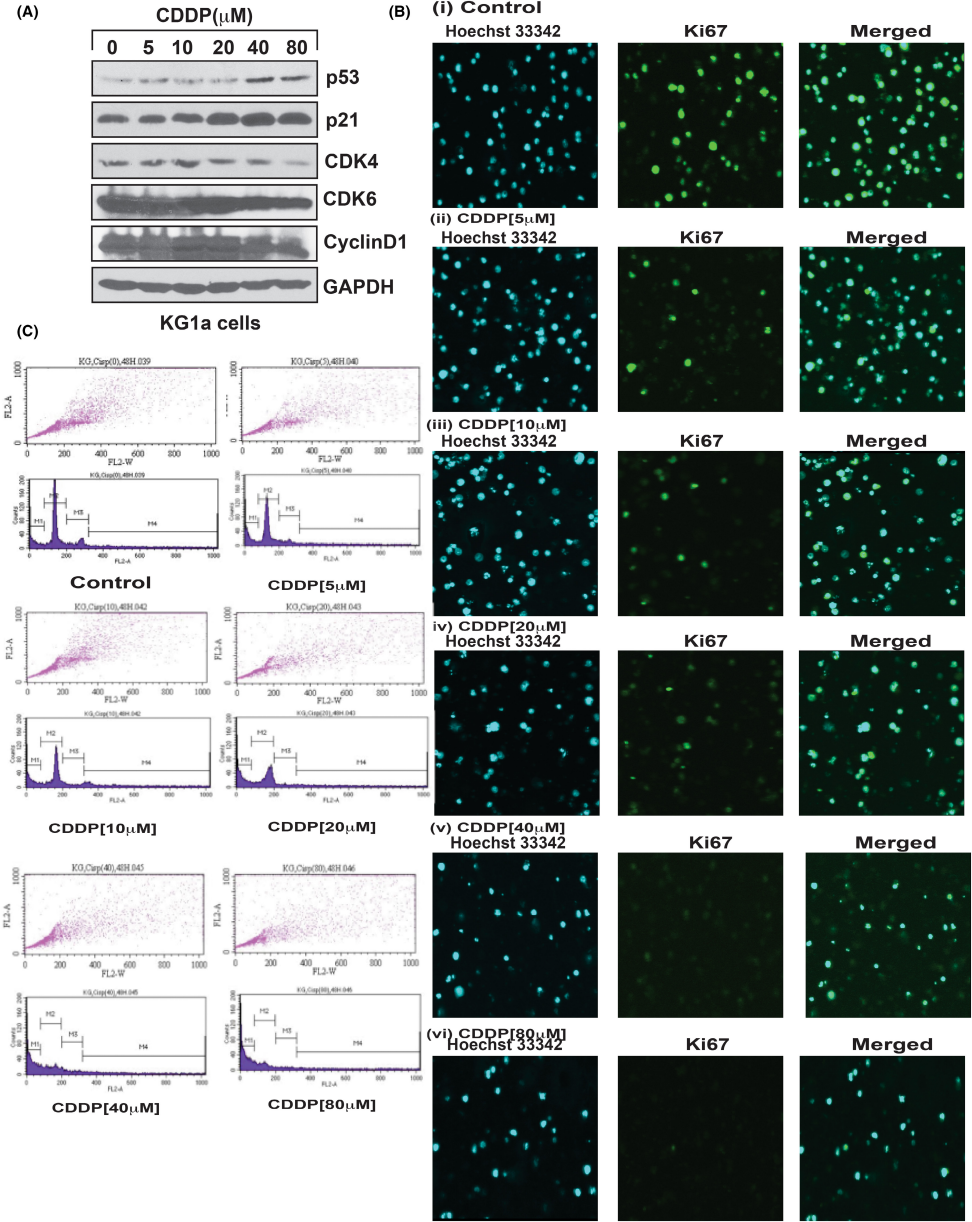
CDDP stimulates p53, inhibits ki67, and causes cell cycle arrest. KG1a cells were treated with different concentrations of CDDP, and the expression levels of p53, p21, cyclins, and cdks proteins were analysed by western blotting. CDDP stimulated p53 and p21 by reduced expression of cyclins (cyclin D1) and cdks (cdk4 and cdk6) in KG1a cells (A). It also reduced the expression of ki67 [B(i–vi)] and induced cell cycle arrest in G1 phase (C) in NB4 cells.

To investigate the effect of CDDP on cell cycle progression, we treated APL cells with different concentrations of CDDP and analysed the expression levels of several proteins including p53, p21, cyclins (cyclin D1), and cyclin‐dependent kinases (CDK 4 and CDK6s) by using the SDS‐polyacrylamide gel electrophoresis technique. We found that the expression of both p53 and p21 was significantly increased (Figure 2A) in CDDP‐treated cells in comparison to control cells without CDDP treatment. On the other hand, we found a significant downregulation of the expression levels of cyclin D1, CDK4, and CDK6 (Figure 2B). Using the immunocytochemistry assay, we also found a significant repression of the activity of Ki67, a cell cycle progression indicator^40^ in CDDP‐treated NB4 cells, relative to its expression in control cells (Figure 2Bi–vi). Taken together, our findings showed that the upregulation of p53 and p21 led to the reduction of Ki67 expression and the arrest of NB4 cells at the G1 checkpoint of the cell cycle (Figure 2Bi‐v,C).

### 3.3 CDDP causes apoptosis

**FIGURE 3:**
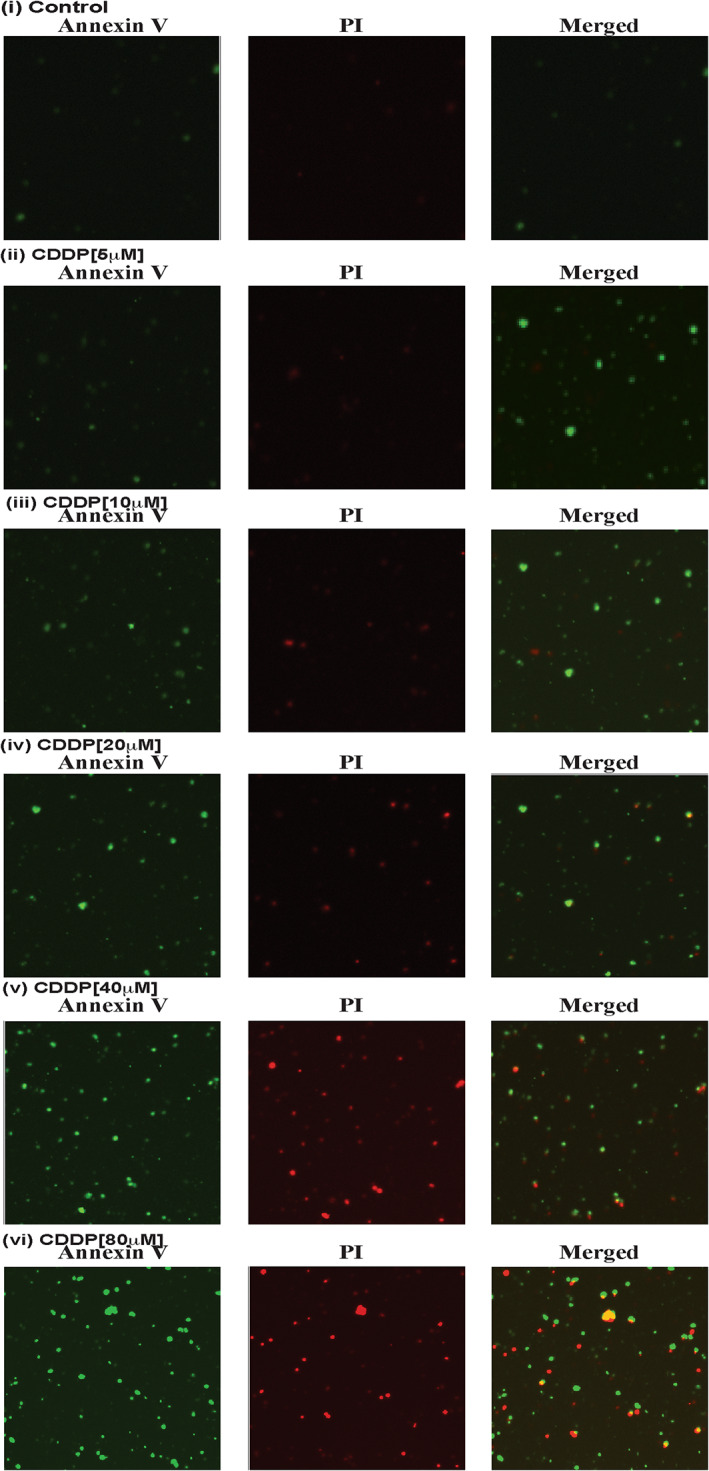
CDDP causes cell apoptosis. NB4 cells were treated with different concentrations of CDDP for 48 h, and both early and late apoptosis were analysed through expression level of both annexin V and PI dye by confocal imaging. CDDP induced both early and late apoptosis in NB4 cells (i–vi).

The potential of CDDP to cause programmed cell death in NB4 cells through the up‐modulation of p53 and p21 expression and arrest of cells at G1 checkpoint was assessed. After treating the cells with CDDP and washing them with cold PBS, early‐stage apoptosis was assessed using a mixture of FITC tag AnnexinV^41^ while late‐stage apoptosis was evaluated using propidium iodine (PI) dye.^42^ Confocal microscopy was performed to characterize the magnitude of Annexin V and PI expression. CDDP‐induced death of NB4 cells was associated with the substantial upregulation of Annexin V and PI expression in treated cells as compared to the control cells (Figure 3i–vi).

### 3.4 CDDP disaggregates MDM2‐DAXX‐HAUSP complex

**FIGURE 4:**
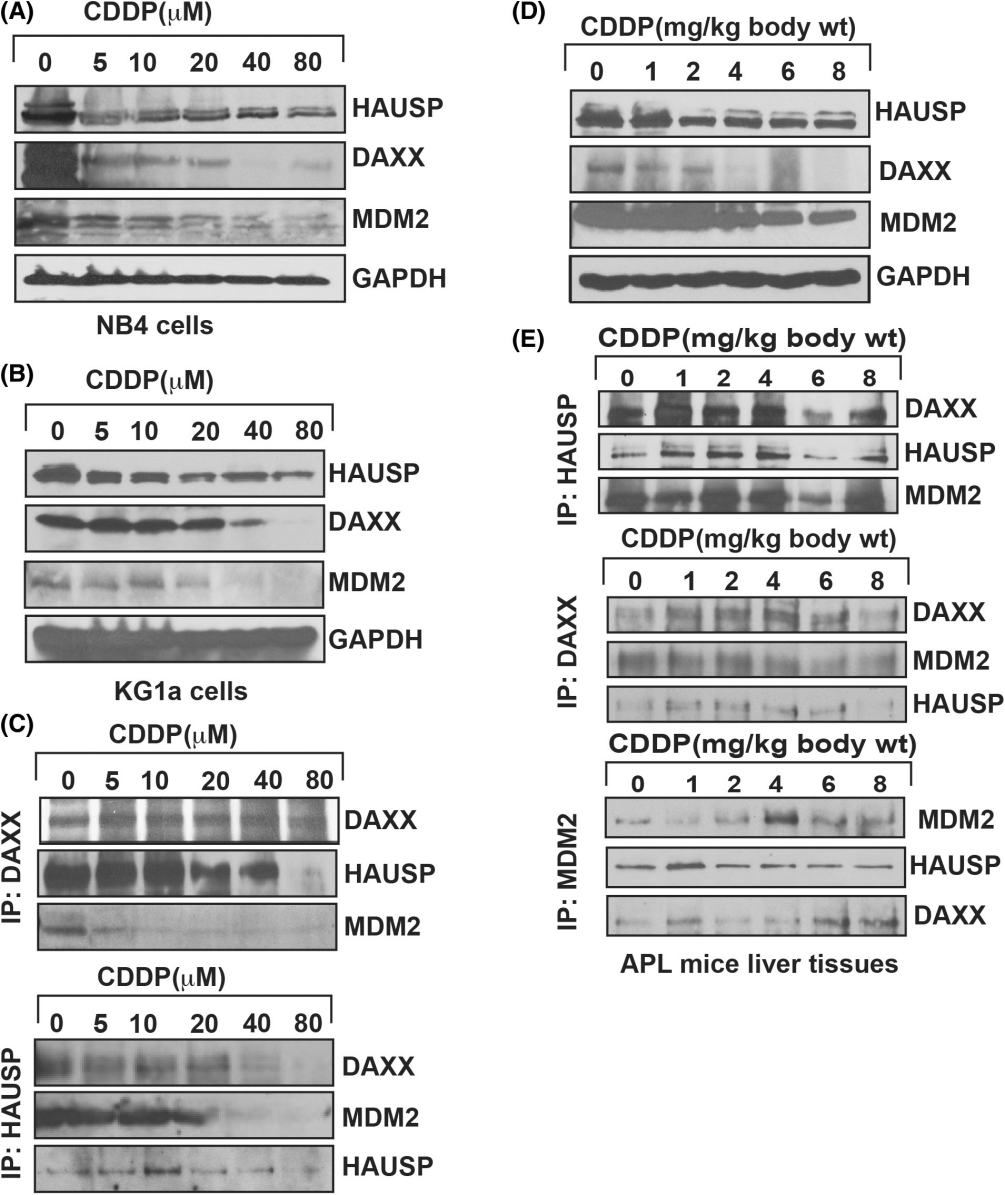
CDDP disrupts MDM2‐DAXX‐HAUSP complex. Both KG1a and NB4 cells were treated with different concentration of CDDP, and complex molecules expression and association were analysed by western blotting and immunoprecipitation (IP). CDDP reduced the expression of complex molecules in both cells (A,B), and molecules were associated each other (C) in KG1a cells. Also, APL mice were treated with different doses of CDDP and complex molecules expression and association were analysed by western blotting and IP. CDDP downregulated the expression of complex molecules (D), and the molecules were associated together (E).

To assess the impact of CDDP on the MDM2‐DAXX‐HAUSP complex, we treated KG1a cells, NB4 cells, and transgenic mice with CDDP and evaluated both the expression and association levels of MDM2, DAXX, and HAUSP by sodium dodecyl sulfate polyacrylamide gel electrophoresis (SDS‐PAGE) and IP. The study result indicated that the expression level of complex molecules was reduced in CDDP‐treated NB4 and KG1a cells (Figure 4A,B) and APL mice liver tissue (Figure 4D). The results from the IP assay showed that CDDP changed the interaction of all complex molecules in both APL cells and liver tissue (Figure 4C,E).

### 3.5 Mechanism of CDDP disruption of complex molecules

**FIGURE 5:**
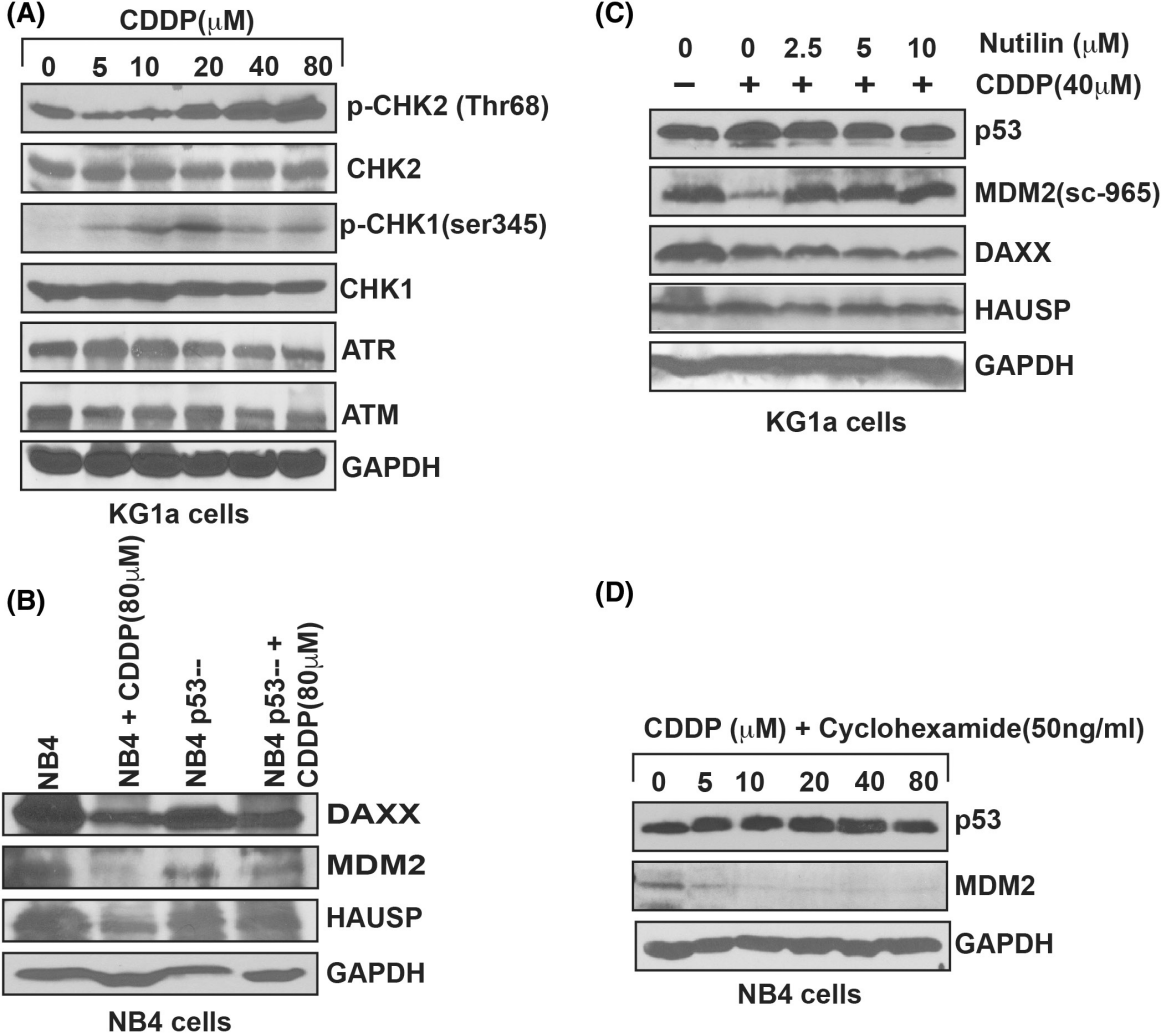
Functional studies of CDDP role in complex disruption. KG1a cells were treated with different concentrations of CDDP, and both expression and phosphorylation of protein kinases (ATM and ATR) and their downstream targets, CHK1 and CHK2, were analysed by western blotting. CDDP reduced the expression of ATM and ATR by stimulating phosphorylation of CHK1 at S345 residue and CHK2 at Thr68 (A). CDDP treatment did not significantly reduce the expression of complex molecules in p53‐knockout NB4 cells (B). Nutilin almost neutralized CDDP‐induced MDM2 reduced expression in KG1a cells (C). P53 and MDM2 proteins turnover was detected in both CDDP‐treated and untreated cells by the pulse‐chase assay in NB4 cells (D).

Cisplatin induced a significant downregulation of MDM2, DAXX, and HAUSP expression by also decreasing the expression of protein kinases (ATM ‐ Ataxia‐telangiectasia mutated, and ATR ‐ Ataxia telangiectasia and Rad3 related ‐ ATR) and activating the phosphorylation of checkpoint kinase 1 (CHK1) and checkpoint kinase 2 (CHK2), respectively, at Ser 345 and Thr 68 residues, in a concentration‐dependent manner in KG1a cells (Figure 5A). Using nutilin‐3 as an inhibitor of MDM2 activity, we assessed its effect on CDDP degradation of MDM2 expression and found that it clearly antagonized CDDP‐induced reduction of MDM2 expression in KG1a cells (Figure 5C). We further assessed the expression level of MDM2, DAXX, and HAUSP in CDDP‐treated NB4 cells in which we knockdown p53 using a lentiviral shRNA approach. We discovered that the expression level of MDM2, DAXX, and HAUSP in p53‐knockdown cells was not significantly reduced, and hence overcoming the action of CDDP (Figure 5B). Also, the data obtained from our protein turnovers assay showed a significant upregulation in p53 half‐life and a significant downregulation in MDM2 half‐life in NB4 cells treated with CDDP (Figure 5D).

### 3.6 CDDP effect in APL mice bone marrow

**FIGURE 6:**
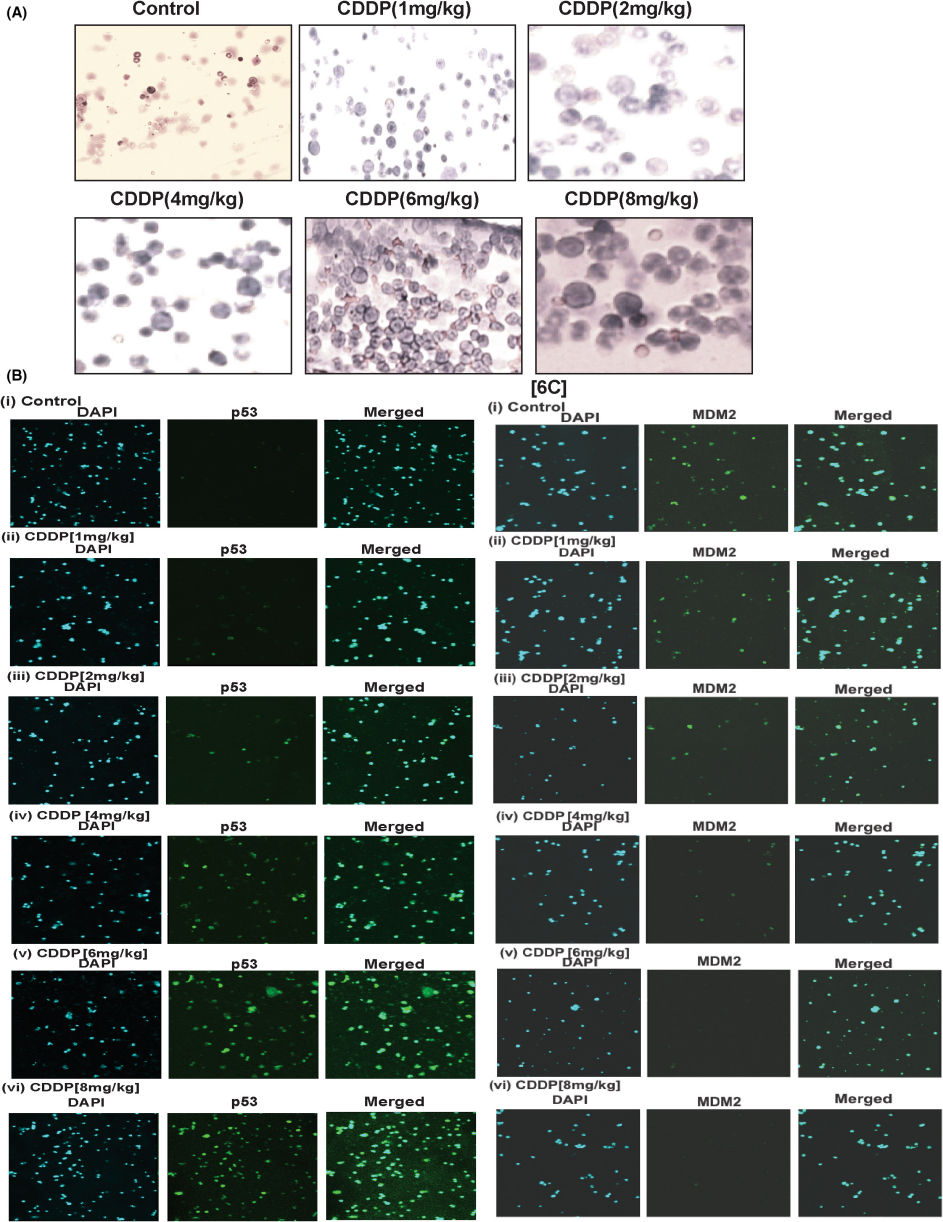
CDDP activates p53 by degradation of MDM2 in mice bone marrow cells. APL mice bone marrow cells were isolated from both control and CDDP‐treated mice, and immunocytochemistry, fluorescence, and confocal imaging were performed to analyse the formation of promyelocytes and the expression levels of MDM2 and p53 proteins. CDDP stimulated the formation of more promyelocytes with dense granules (A) and activated p53 (B), leading to the degradation of MDM2 (C) in bone marrow cells.

For investigation of CDDP effect on APL mice bone marrow, APL mice were treated intraperitoneally for 3 days with different doses (0, 1, 2, 4, 6, and 8 mg/kg body weight) of CDDP and the femur and tibia‐fibula bone marrow cells were isolated. Thin smears of these bone marrow cells were made on slides, air dry, fixed with phosphate buffer, stained with Wright‐Giemsa staining, and imaged by fluorescence microscopy. The study results showed that CDDP treatment significantly increased the number of promyelocytes with dense granules in a dose‐dependent fashion (Figure 6A). We also made protein lysates of bone marrow cells of both control and CDDP‐treated animals and checked the expression profiles of p53 and MDM2 by immunocytochemistry and confocal imaging. Interestingly, we discovered that the expression level of p53 was upregulated while the expression level of MDM2 was downregulated in a dose‐dependent manner (Figure 6B,C) in bone marrow cells.

### 3.7 CDDP effect on APL mice liver

**FIGURE 7:**
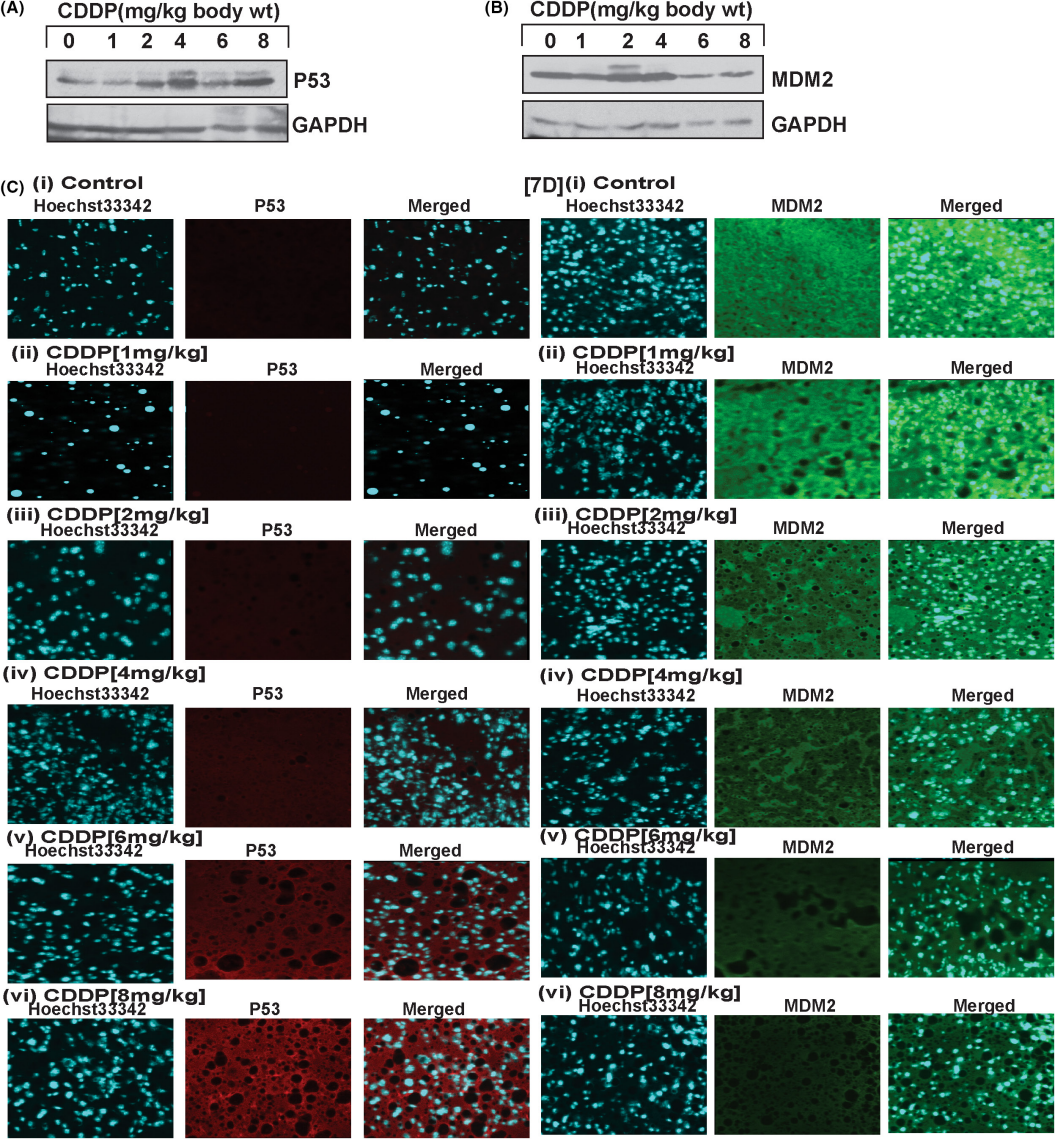
CDDP stimulated p53 by degradation of MDM2 in liver tissue of APL mice. Both control and CDDP‐treated APL mice livers were collected, and both protein lysate and cryosections (5 μM) were made simultaneously. Expression of both p53 and MDM2 in protein lysate was measured by western blotting (A,B) and by immunohistochemistry and fluorescence microscopy of cryosections (C,D).

We made protein lysates and cryosections (5 μM) of liver tissues from both control and CDDP‐treated APL mice. We then assessed the expression levels of p53 and MDM2 by SDS‐PAGE and IP, and fluorescence microscopy. The study results showed that CDDP activated p53 activity by downregulating MDM2 expression in a dose‐dependent manner (Figure 7A–D).

## 4 DISCUSSION

Cisplatin is an anti‐cancer drug (7) that has been widely used for the cure of various types of human cancer.^8^ It enhances toxicity of ATO in many cancer cells including squamous cell carcinoma of the head and neck,^9^ small‐cell lung cancer,^10^ ovarian cancer cells,^43^ oral squamous cell carcinoma cells,^44^ and chronic myelogenous leukaemia (CML) cells.^45^ p53, a tumour suppression protein, modulates cell cycle progression, genomic integrity, and programmed death in cells that are exposed to various stress conditions. It has been reported to be mutated or deleted or suppressed in several human cancers.^13^, ^29^ Reactivation of p53 has a great potential as a novel strategy for the cure of human cancers.^30^ P53 is a novel target of therapeutic strategy for APL patients, as it widely modulates APL cells growth, cell cycle regulation, and apoptosis.^17^, ^18^, ^19^, ^20^ CDDP induces cytotoxicity,^2^ inhibits cell growth through cell arrest, and induces DNA alteration, oxidative damage, and programmed death of APL cells.^23^, ^24^, ^25^, ^26^ However, its specific mechanism of action on p53 activation, cell cycle modulation, and apoptosis are not fully elucidated. Published studies have pointed out that ATO inhibits both the growth and progression of cell cycle in APL cell lines.^4^ In the present research, we found that CDDP significantly inhibits the proliferation of HL‐60, KG1a, and NB4 cells in a concentration‐dependent manner (Figure 1A–C). Scientific evidence suggests that both ATO and CDDP slow down the normal process of cell cycle by upregulating the activity of p53 and p21 in APL cell lines.^2^, ^4^, ^23^ Our findings indicate that CDDP stimulates the expression of p53 and p21 by downregulating the expression of Ki67, cyclins, and CDKs, leading to cell cycle arrest mostly at G1 phase in NB4 cells (Figure 2A–C). It has been reported earlier CDDP induces apoptosis in HL‐60 cells.^23^, ^24^, ^46^ We have demonstrated that CDDP causes both early and late apoptosis in NB4 cells through stimulation of expression of Annexin V and PI simultaneously (Figure 2Di–vi). Accumulating evidence suggests that p53 is activated inside the cancer cells due to oxidative stress, DNA damage, MDM2‐DAXX‐HAUSP disruption, and downregulation of MDM2 expression.^2^, ^4^, ^13^, ^14^, ^15^, ^16^

From our previous study, we found that ATO activates p53 through a downregulation of complex molecules in the tissues of APL mice.^47^ Our new findings reveal that CDDP treatment significantly disrupted MDM2‐DAXX‐HAUSP and downregulated the association and expression of its constituents/molecules in NB4 cells, KG1a cells, and APL mice liver tissue (Figure 3A–E). Several other studies have provided evidence that DNA damage and stress signal is transmitted from protein kinase (ATM and ATR) and its downstream CHK1 and CHK2 target phosphorylation at different residues in cancer cells.^4^, ^13^, ^48^, ^49^, ^50^ Our research data indicate that CDDP‐induced stress signal is catalysed by the reduced expression of protein kinases (ATM and ATR) and stimulation of substantial phosphorylation of CHK1 at Ser345 residue, and CHK2 at Thr68 residue in KG1a cells (Figure 4A). Additionally, the results of our functional studies demonstrate that CDDP‐induced repression of complex molecules was not significantly observed in NB4 cells in which p53 was knock‐down using the lentiviral shRNA approach (Figure 4B). Moreover, we found that nutilin‐3 treatment almost overcame/neutralized CDDP‐induced downregulation of MDM2 expression in kG1a cells (Figure 4C). Also, CDDP stimulated p53 turn‐over by downregulating MDM2 protein turn‐over in KG1a cells (Figure 4D). Our findings also show that CDDP treatment triggered more promyelocytes with dense granules, activated p53 expression, and downregulated MDM2 expression in bone marrow cells (Figure 5A–C) and liver tissues (Figure 6A–D) of APL mice. Furthermore, CDDP activation of p53 was mediated via a stress signal transmitted via protein kinases (ATM, ATR) and their downstream targets, leading to MDM2‐DAXX‐HAUSP disruption, cell cycle arrest, and APL cell apoptosis. Taken together, the findings highlight a potential target that may be used in new APL drug designing.

The large body of novel literature on APL articulates that APL is M3 subtype of AML formed inside bone marrow due to chromosomal mutation usually between chromosomes 15 and 17. This mutation results in the formation of fusion oncogene, promyelocytic leukaemia (PML)‐retinoic acid receptor‐α (RARa), leading to the production of RARa‐PML. PML‐RARa is responsible for pathogenesis of APL while RARa‐PML would be important molecular marker used for monitoring of APL patients.^3^, ^4^ APL is initiated through PML‐RARa by blocking differentiation and self‐renewal of leukaemic progenitor cells. PML‐RARa protein represses both RARa and non‐RARa target genes and disrupts PML nuclear bodies.^51^ Initially, APL patients were treated with life‐threatening chemotherapy with 6‐mercatopurine (6‐MP), methyl‐glyoxal guanyl hydrazine, daunorubicin (DNR), and cytarabine.^52^, ^53^ The treatment for APL patients was revolutionized by all‐trans retinoic acid (ATRA) and ATO with induction of complete remission (CR) rate reported at 72%–85% worldwide.^54^, ^55^, ^56^

Recent success of APL patients has shown that retinoic acid (RA) cures low‐risk patients and may cause life‐threatening complication of ATRA syndrome. Also, ATO has been used alone. However, the combination of both ATRA and ATO has been used successfully for treatment of most APL patients.^51^ ATRA‐ATO is recommended for treat low‐ and mild‐risk APL patients without ATRA syndrome with improved CR rate of around 90–95% and reduced relapse rate.^57^ However, it does not work properly in high‐risk and RA‐resistant promyelocytic leukaemia zinc finger (PLZF)‐RARα APL patients and may cause some sort of cardiovascular problems. ATO is recommended for high‐risk patients, but recently, resistance has been reported in PLZF‐RARα APL patients worldwide.^4^ It has also been reported clinically that RA, anthracycline, and ATO combination cures APL patients up to 90%.^51^

P53 expression is reduced by MDM2 or mutated in most of cancer cells including APL. ATO activates p53 through degradation of MDM2, arrests cell cycle, and forces APL cells to undergo apoptosis.^4^ It directly binds PML by oxidation‐triggered disulphide bond formation, leading to PML sumoylation, ubiquitylation, and proteasome‐mediated degradation.^51^ ATO also enhances PML nuclear body assembly in APL cells.^19^ Low dose of RA induces APL cells differentiation, but high dose of RA degrades PML‐RARa and induces PML‐nuclear body reformation and PML‐transformation related protein (Trp53) formation, leading to eradication of leukaemia‐initiating cells by senescence but not apoptosis in bone marrow of mice model APL.^19^, ^51^

Recently, ATO‐resistant APL patients having RA‐resistant promyelocytic leukaemia zinc finger (PLZF) RARα have been reported worldwide. Existing evidence suggested that cisplatin (CDDP) enhances toxicity of ATO in many cancer cells including squamous cell carcinoma of the head and neck, small‐cell lung cancer, ovarian cancer cells, oral squamous cell carcinoma cells, and CML cells.^9^, ^10^, ^43^, ^44^, ^45^ Moreover, CDDP induces cytotoxicity in APL cells through oxidative stress, DNA damage, stress signalling pathway.^2^ CDDP, a platinum‐containing drug, may be used as an ATO alternate drug for ATO for treatment of APL patients after clinical trials have been conducted and the pharmacological and toxicological data have been thoroughly evaluated.

Although further biomedical research will be needed in this regard, novel findings also provide a scientific basis for further considerations of CDDP as a potential therapy for APL patients. Our findings clearly indicate that CDDP activates p53, arrests cell cycle, and forces APL cells to undergo apoptosis. We have also demonstrated that CDDP downregulates the expression of DAXX, HAUSP, and MDM2 proteins leading to the upregulation of p53 protein. In mechanistic terms, CDDP‐induced cellular stress (oxidative stress, DNA damage)^2^ is transmitted through signal transduction by protein kinases (ATM and ATR) and their downstream targets, CHK1 and CHK2 phosphorylation at different residues, leading to activation of p53 in APL cells. Hence, these mechanistic aspects of CDDP mode of action are very important in fostering the translation of novel findings into care of APL patients.

## AUTHOR CONTRIBUTIONS

**Sanjay Kumar:** Conceptualization (equal); formal analysis (equal); investigation (equal); methodology (equal); validation (equal); writing – original draft (equal); writing – review and editing (equal). **Paul B. Tchounwou:** Conceptualization (equal); data curation (equal); formal analysis (equal); funding acquisition (lead); methodology (equal); project administration (lead); resources (lead); supervision (lead); validation (equal); visualization (equal); writing – original draft (supporting); writing – review and editing (lead).

## FUNDING INFORMATION

This research was financially supported by National Institutes of Health NIMHD Grant No. U54MD015929, through the RCMI‐Center for Health Disparities Research at Jackson State University, Jackson, MS, USA.

## CONFLICT OF INTERESTS

The authors declare that they have no competing interests.

## Data Availability

The data that support the findings of this study are available from the corresponding author upon reasonable request.
